# Double‐Layer Masking of Suffering After Pregnancy Loss: A Grounded Theory Study from a Male Perspective

**DOI:** 10.1111/jmwh.13353

**Published:** 2022-03-11

**Authors:** Sara Fernández‐Basanta, Carmen Coronado, María‐Jesús Movilla‐Fernández

**Affiliations:** ^1^ Research group GRINCAR, Department of Health Sciences, Faculty of Nursing and Podiatry, Campus Industrial of Ferrol University of A Coruña Ferrol 15403 Spain

**Keywords:** emotional adjustment, men, miscarriage, qualitative research, stillbirth

## Abstract

**Introduction:**

Men can express different responses after pregnancy loss. This loss can interfere with their expectation of parenthood, new life, and future hopes. Expectations from the social construction of gender can encourage them to maintain an image that contradicts their actual feelings. This can lead to isolation, distancing, and difficulties in seeking support. The scarcity and low representation of men in previous studies makes research that captures the complexity of their experience necessary. The aim of this study was to explore how men confront the suffering caused by pregnancy loss.

**Methods:**

This study is part of a larger research project focusing on the experiences of parents and midwives following pregnancy loss. In this study, 22 cisgender and white heterosexual men who experienced pregnancy losses participated in semistructured interviews. Data were analyzed iteratively using constructivist grounded theory methods.

**Results:**

The substantive theory of double‐layer masking of suffering emerged as way to explain the confrontation of suffering after pregnancy loss from the male perspective. The themes, (1) suffering beyond physical loss, (2) rationalization in the search for meaning, and (3) keeping a façade with others, show the impact that this loss had on men, which was masked by the meaning they gave to the situation and by its social expression.

**Discussion:**

The findings provide a theoretical conceptualization of the masking these men use to deal with the suffering they experienced from this situation. These aspects provide reasons for including these individuals in the assistance given by midwives after a pregnancy loss. Collaboration between specialized and primary care, along with staff training and support, is necessary for the provision of couple‐centered care after pregnancy loss.

## INTRODUCTION

Pregnancy loss, including miscarriage and stillbirth, is a relatively common occurrence. Although difficult to exactly quantify,^1^ it is estimated that 20% to 30% of pregnancies end in miscarriage, or approximately 23 million miscarriages worldwide per year. The global rate of stillbirths is 13.9 per 1000 total births, or approximately 2 million stillbirths per year. However, these statistics do not represent the totality of losses.^1^, ^2^, ^3^, ^4^, ^5^

Pregnancy loss sparks different, dynamic, and highly individualized responses in parents.^6^ Some may feel guilt and shame, others relief and hopefulness about the future, or even ambivalence about pregnancy and loss.^7^ However, this loss interrupts the dream of parenthood, a new life, and future hopes.^8^ Outward expressions of suffering do not necessarily indicate what an individual is experiencing or what they need. These expressions may be influenced by several factors.^9^ The social context encourages men to keep a façade of stoicism or a strong and protective role and does not allow them to express their emotions to others, especially to their partner.^10^ Additionally, there is a misunderstanding and lack of social recognition of their suffering. Under this social construct, the woman's suffering is considered more legitimate than their own, which favors isolation and distancing of the man, thus making it more difficult to elicit support.^11^, ^12^, ^13^, ^14^

**QUICK POINTS**

✦ Pregnancy loss implied more than a physical loss for men because their desire to become a parent was frustrated, and the woman's suffering was added to their own.
✦ Men masked suffering by rationalizing the meaning‐searching process.
✦ Avoidance behaviors, which led to further suffering, were used to hide their feelings.
✦ The theory of double‐layer masking of suffering suggests the need for a comprehensive and couple‐centered approach to pregnancy loss, collaboration between specialty and primary care, and the involvement of educators and managers.

Research that centers on men's experiences is necessary to capture its complexity, which would allow the design of support recommendations and delivery systems that are custom‐tailored to their needs.^15^, ^16^ The literature has mainly focused on the experiences of heterosexual cisgender women^17^ rather than those of men and other partners.^11^, ^18^

Cultural factors are important elements to consider within the social construction of death.^8^, ^21^, ^22^ In the Spanish context, studies have mixed the experiences of women and men because of the limited participation of the latter.^19^, ^20^ In Spain, a model predominates in which death is constructed under the hegemony of medical discourse and is confined to private spaces, and individual attitudes are focused on conscious denial and rejection of emotions. However, traditional values and beliefs surrounding death have a strong influence on personal consciences.^23^ Therefore, the aims of this article were to explore how men confront pregnancy loss and to develop an empirical model.

## METHODS

### Research Design

The methodology of this qualitative study was grounded in a constructivist theory developed by Charmaz (2006).^24^ The constructivist paradigm recognizes the existence of multiple social realities (relativism) and positions the researcher in a key role in the construction of theory (subjectivism). Through reflexivity, the researcher becomes an actor and instrument of the research.^25^ This report complied with the standards for reporting qualitative research.^26^

The research team consisted of trained qualitative researchers with experience in researching pregnancy losses from the perspective of parents and nursing staff. There was no previous relationship with the participants.

### Sampling and Participants

This study was part of a larger research project, designed and developed by the authors, that investigated the experiences of parents and nursing staff after pregnancy loss in urban and rural settings of northwestern Spain. The recruitment of participants involved purposive sampling. Individuals were eligible to participate if they were female‐ or male‐identifying, coupled or single, had experienced a pregnancy loss (eg, miscarriage, termination due to fetal anomalies, or stillbirth), and belonged to a health care service area in northwest Spain. Midwives and gynecologists collaborated in participant recruitment by distributing an informational flyer that requested a participant's consent to be called by the research team. After giving consent, the participant was contacted and an appointment scheduled at their convenience.

### Data Collection

S.F.B. or M.J.M.F. conducted in‐depth, semistructured couple interviews with all participants (Table 1), asking questions about both individual and shared experiences. The reason for conducting joint interviews was to elucidate tacit knowledge of their experiences by prompting, clarifying, and making explicit men's assumptions and feelings.^27^ The interviewer asked further questions as needed to encourage deeper explanation of responses. Field notes collected after the interviews were integrated into the transcripts to enrich the data. The interviews were conducted between 2015 and 2019 in Spanish and Galician and mostly carried out at the participants’ homes but sometimes in an office. The average duration was 90 minutes, and they were tape recorded and transcribed by S.F.B. Anonymity was guaranteed, and audio recordings were destroyed using acceptable industry procedures.

**Table 1:** Semistructured Interview Script

**Thematic Field**	**Examples of Questions**
**Contextualization of pregnancy loss**	How has it happened? What did you feel? What has this loss meant for you?
**Health support**	What role did health care professionals play?
**Family and social support**	What role did your family play at that time? And your social environment? And now?
**Returning home**	How was the return home? Has your routine and family life changed?
**Dealing with loss**	How do you deal with the loss? Have you sought help on your own? What type?
**Experience for the couple**	What has this experience meant for you as a couple?
**Experience at the time of the interview**	How do you feel now? Do you keep any mementos? Have you performed any farewell rituals?

### Ethics

The study obtained approval of the Autonomous Committee of Research Ethics of Galicia (registration code 2015/232) and had health area access permission from Ferrol, Spain. All participants received written and oral information about the study and its voluntary nature, as well as the assurance of confidentiality. Informed consent was obtained before the interviews.

### Data Analysis

After transcription of the interviews, initial coding was performed line by line and incident to incident. Codes were named as gerunds and were kept short, precise, analytical, and close to the data. By comparing data, the focused code was developed. Memos were created during data collection and analysis to record analytical thoughts and insights. Axial coding, which consisted of clustering codes with similar content into concepts with a higher abstraction level, was then performed. With this process, provisional analytical categories were established. After the first 15 interviews, an initial framework was developed to organize the emerging categories. To deepen the development of the properties of these categories, the researchers used theoretical sampling. At this point, data were not restricted to the first weeks after the loss: this allowed the categories to be tested and saturated. The coding was based on an abductive process. The categories were considered saturated when gathering new data no longer provided new theoretical insights or revealed new properties. Ultimately, the analysis moved toward a conceptual framework that consisted of 3 main topics.

The criteria outlined by Charmaz^24^ included credibility, originality, resonance, and usefulness. Throughout the investigation, the research team met periodically to review the coding, discuss the emerging analysis, and resolve inconsistencies. The iterative decision‐making process during the research was collected in memos. The emerging theory was presented to midwives before publishing, which helped to verify resonance and recognizability. Quotations taken from interviews were identified by participant number and the weeks’ gestation at which the loss occurred. An English native speaker in collaboration with the first author translated the quotations.

## RESULTS

The study comprised 22 cisgender and white heterosexual men who experienced 23 pregnancy losses, as one participant experienced a second fetal death after the first interview and was interviewed twice. Table 2 presents the sociodemographic characteristics of the participants.

**Table 2:** Sociodemographic Characteristics of Participants (N = 22)

**Characteristic**	**Value**
**Sociodemographic Characteristics**	
**Age, mean (SD), y**	37.5 (6)
**Educational level, n (%)**	
Basic education	6 (27)
Secondary education	2 (9)
Professional training	10 (45)
Higher education	4 (18)
**Employed, n (%)**	18 (82)
**Race and ethnicity, n (%)**	
White	22 (100)
**Relationship status, n (%)**	
In a relationship	22 (100)
**Pregnancy Loss Characteristics**	
**Type of loss, n (%)**	
Miscarriage	17 (74)
Stillbirth	5 (22)
Induced abortion for fetal anomaly	1 (4)
**Weeks gestation when the loss occurred, mean (SD)**	15.3 (10)
**Previous loss, n (%)**	4 (18)
**Previous children, n (%)**	7 (32)
**Other Characteristics, n (%)**	
**Insemination procedure for infertility**	1 (4)
**Ovarian stimulation for infertility**	1 (4)
**Pregnant during the interview**	2 (9)
**Subsequent pregnancy after the pregnancy loss**	1 (4)
**Time between Loss and Interview, Mean (SD), wk**	15.8 (36)

**Figure 1:**
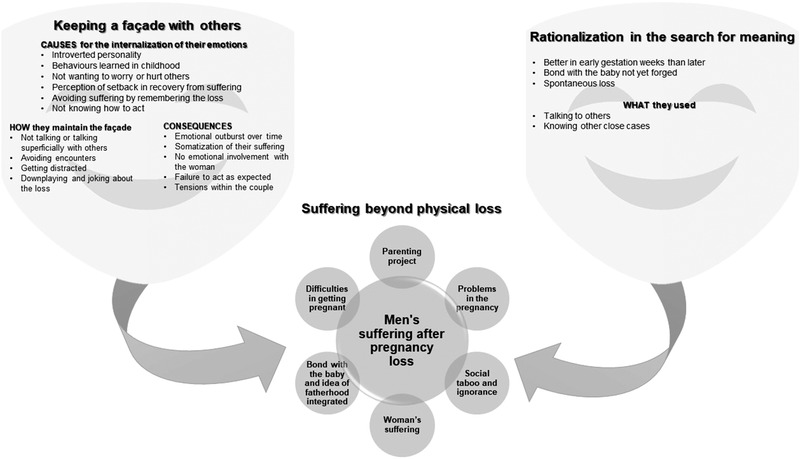
Double‐Layer Masking of Suffering

The double‐layer masking of suffering model shows how men struggle against the suffering caused by a pregnancy loss (Figure 1). The central axis of this model shows the suffering that men experienced, based on the theme of suffering that goes beyond physical loss. This was conditioned by several elements such as frustration in parenthood, woman's suffering added to their own, and the bond with the fetus during pregnancy. Men were especially affected by the loss of a desired pregnancy. However, when there were problems during the pregnancy, the loss was a relief by exonerating them from making difficult decisions later.

Under these circumstances, men tended to mask their suffering more than women. Rationalization in the search for meaning showed the process of integrating loss into their life by transforming it into pseudoreasonable feelings (ie, thoughts or behaviors that would otherwise cause greater suffering). This rationalization process was influenced by partners, health care professionals, and the social environment. Keeping a façade with others was another layer of masking in the social expression of their suffering. Not talking, talking about the case superficially with others, avoiding encounters, getting distracted, or downplaying and joking about the loss were some examples. This may have led to emotional outbursts, somatization, becoming emotionally distant from their partner, or feeling that they failed in their expected role and caused tension in their relationship.

### Suffering Beyond a Physical Loss

The news of pregnancy loss, and even the communication of problems in the development of the pregnancy, came as a shock and caused an emotional collapse. This was because, in many cases, there were no previous signs or symptoms indicating this outcome, or because some men were unaware that pregnancy losses could occur after 12 weeks’ gestation. In late pregnancy losses, the idea of fatherhood had already integrated into their lives, for example, by knowing the sex or having chosen the name, which led to greater shock.

However, for parents who had already been informed of pregnancy issues, the loss was not as traumatic but was experienced with some relief by ending their worry. The spontaneous pregnancy loss was understood to have a lower physical risk for women than a potentially necessary abortion. “It was something like… Not a confirmation, but almost releasing. I do not know how to explain it to you. ‘Well, that's it. That's it. Stop thinking about it’” (#9, 15 weeks).

Several men reported that pregnancy loss was emotionally difficult, especially in cases in which the presence of the fetus was evident. For many, it also meant frustration regarding fatherhood, which was evidenced by feelings of disappointment and envy when seeing other pregnant couples or newborns. In most cases, pregnancies were desired, and for some, the pregnancy came with many difficulties. For those who were surprised by pregnancy, they eventually became excited about it.

Men conveyed a concern for their partner and the physical suffering experienced in the abortion process and subsequent discouragement. Feelings of emptiness, suffering, sadness, and fear were common. In addition, the loss made them aware of potential future pregnancy issues. “The first [pregnancy loss] was less harsh. But this second one… you think too much about it, you think many things. You have a hard time” (#7, 7 weeks).

### Rationalization in the Search for Meaning

Finding meaning from grief was strongly characterized by rationalization. Many men expressed that the loss was the best outcome when compared with others, such as abortion. This comparison was made according to their personal situation and, in many cases, when the loss could not be explained. For example, having children prior to the loss, or when the abortion was quick or did not imply physical suffering for the woman, were some examples of these comparisons. In many cases, the lack of explanation for the loss led to a search for a culprit, as this man expressed: “I believe that there was some negligence at the hospital. We have talked about it. She was not well. In fact, when she was discharged, she was not quite well” (#21, 35 weeks).

The fact that the loss occurred during pregnancy, and especially in the early weeks, was understood as advantageous compared with happening later. Early losses were characterized by a lack of tangible elements to prove the existence of pregnancy and, therefore, of their future fatherhood. For surprise pregnancies, the idea of imminent fatherhood was still in development and not yet internalized. In many cases, the time between breaking the news of pregnancy and the loss was very short.

The feelings of attachment and bonding during pregnancy also influenced the meaning they gave to the loss. For some, a short period of gestation was not enough to establish a strong bond, thus diminishing the emotional impact of the loss. However, for others, it was experienced as the loss of a child, which can be explained by the bonding established in a planned pregnancy.

It was also understood that when faced with problems in fetal development, an involuntary pregnancy loss could be the best option, better than abortion, as this man reported: “It's good news, because, under these circumstances, if something is wrong, and you have to wait until 16 weeks of gestation to abort […] Here is when you have nothing to do anymore” (#13, 14 weeks).

Even in the case of elective abortion, the health care professional's explanation of the noncompatibility with life helped because the decision meant ending the fetus's suffering, which alleviated personal suffering.

This approach helped men to relativize the issue and, therefore, to find comfort in this perspective. Occasionally, conversations with their partner, relatives, friends, or health care professionals helped them to understand the loss in this way. Knowing other similar cases in which couples went on to full‐term pregnancies also gave them hope. Furthermore, this also helped them understand that they were not alone, which lessened their own loss. They finally accepted that loss is a natural and common event.

In advanced gestational losses, experiential or material memories contributed to the existential meaning of the newborn. Initially, men did not want to see or hold the child after birth. The fear of its physical appearance, the pain of the recollection, or the lack of time to make the decision were some of the causes. For some, this rejection caused regret after the decision. However, those who did see the newborn reported that it was a way to confront suffering. Mementos of the loss, such as photos or footprints, were considered painful, injurious, macabre, and unnecessary reminders. They refused to get tattoos related to the loss or to organize farewell events partly because they perceived that the social environment did not understand the loss. However, ultrasound images, maternity pictures or videos, or even pregnancy tests were kept because of the happiness of remembering those moments.

### Keeping a Façade with Others

The emotional management of suffering after pregnancy loss was characterized by internalization of emotions. Men reported that suffering had to be managed alone or with the partner. This perception was explained by introverted personality and behaviors learned in childhood. This is why some felt displeasure or discomfort when expressing their feelings.

It was psychological suffering along with my sadness. And as it happens to me with, and other things in life, each one of us feels in our way, and I tend to hide emotions. Well, with more reason, I thought that I better hide them, because she already had enough with her physical suffering to see me suffer. So, I still think that I strayed further than I was supposed to. (#20, 10 weeks)

The men were able to show a façade of unreal integrity because showing their feelings would imply a setback in their overcoming the loss and an emotional collapse of the woman.

The emotional bond with the fetus was most deeply rooted in the woman. Therefore, she was the one who suffered the most, and physical pain was added to the emotional suffering. For many men, the emotional suffering of the woman was the central axis of their own suffering. For this reason, they were able to convince themselves to be emotionally strong, which meant repressing their emotions and maintaining an attitude of constant encouragement with their partner. They tried to show support by talking to their partners with an optimistic approach to giving meaning to the loss or to eliminate any guilt that they might experience. They also provided time and space for their partner to vent. Nonetheless, many men showed their support by not talking about the loss or distracting their partner. In part, this was also because confronting the loss agitated the men emotionally, and they felt that reminders of the loss would hurt themselves even more. The emotional expression of their partner's suffering also elicited the men's suffering, and they reported not knowing how to act when they saw their partner in distress.

This avoidance also carried over to not talking to others about the loss. In advanced losses, in which the existence of the fetus was known to others, some men changed their routine to avoid the questions of others. For example, men did their grocery shopping in other cities or took time off work in lieu of paternity leave. Questions and comments from others could be damaging, as well as remind them of the loss.

To confront the suffering, most men tried to avoid thinking about it and yearned to go back to their life before the loss. To reach this goal, returning to work or distracting themselves with leisure activities allowed them to put aside constant thoughts about the loss, although these thoughts could return at night. Among the distraction activities, they highlighted going out, playing football with friends, playing board games, watching television, or reading. Moreover, for those men who already had previous children, they had an important source of motivation to struggle against the suffering. Their role as parents forced them to recover, not think about the loss, and be cheerful with their living children.

In the weeks after the loss, many men reported that they stopped talking about it. However, some continued to discuss it by trivializing and joking about the loss. Many of them only felt that they could speak with their wife because their social network was unfamiliar with the pregnancy. In cases in which people knew of their future paternity, they generally did not hide the loss but avoided bringing it up. They talked superficially about the loss and showed emotional detachment. Some men reported difficulties in discussing it because it involved remembering what happened and they feared the reaction of others, even when the other person had been through a similar loss. In many cases, the repression of emotions resulted in emotional outbursts over time. Some of them may have even somatized their suffering and experienced physical symptoms.

Some men felt that they did not fulfill their partner's expectations and reported that the way they dealt with their suffering could have contributed to becoming emotionally detached from her. The dissonance regarding how the loss was confronted could trigger tension in some instances, as shown in this excerpt:

I've come to ask her a couple of times… “Please change, if not this… We ended up separating. I just can't […] I've also lost a child [too]”. I told her that she was taking advantage of the situation, with a person who is suffering like her, or almost. (#21, 35 weeks)

## DISCUSSION

Our results show the influence of pregnancy loss on men, and the theory of *double‐layer masking of suffering* (Figure 1) emerged as an explanation of the struggle against suffering. Men masked suffering by rationalizing the meaning‐searching process and by hiding their emotions in their encounters with others.

In caring science, suffering is one of the major ontological concepts and relates to a human being's struggle between good and evil in a state of becoming. According to Eriksson et al,^28^ there is meaning in suffering, and it emerges when a person reconciles their self with the situation and then finds possibilities and meaning. However, this growth can be altered if the person denies suffering and its possibilities. Our results show that the pregnancy loss entailed suffering for men, not only because of the physical loss but also because of the nonfulfillment of becoming a parent. Preparing for parenthood and bonding with the child begins during pregnancy.^29^ However, bonding can be influenced by planned pregnancy, the quality of the relationship with the woman, the gestation week, and the sociocultural context.^30^, ^31^ This could explain why some men adapt and become attached to fatherhood when pregnancy begins to show in the woman's body.^32^ This, together with the existence of previous problems in pregnancy, the woman's suffering, and difficulties in getting pregnant, modulated their suffering.

Men responded by masking and belittling their suffering. They tried to find meaning in the loss from a position that did not contemplate the suffering, but instead tried to look for a cause. This is in line with the experiences of other men, in which the search for meaning is based on finding a biological explanation that justifies what happened.^12^, ^33^ Our results describe the meanings they found and how they elaborated on them.

This need to explain the events that happened is recognized by Eriksson et al.^28^ However, trying to uncover the answer to *why* can lead to more suffering. The men in our study faced loneliness and feeling unwelcome in their quest to understand. The men lacked spaces in which to confront their suffering, as socially they felt that they were not allowed. In many cases, there were no interventions that focused on them to help alleviate their suffering. Relieving a person's suffering first requires that it is recognized, and then they should be given time and space to reconcile with it.^34^

Our findings show a strong influence of the social construction of gender in how men confront and express suffering. Contemporary masculinity has often been understood as unemotional, emotionally impaired, or stoic, which is seen as a consequence of Western gender norms.^35^ Men are associated with reason, and all stereotypical feminine characteristics and qualities are stigmatized, including openness and vulnerability.^36^ In addition, men are expected to control insecurity and other feelings.^37^ Men's emotional incompetence and cultural expectations to be stoic may make them too emotional but in the wrong way.^38^, ^39^ This may have contributed to the men in this study focusing on their partner, rather than on their own suffering.^11^, ^14^, ^40^ The literature indicates that men tend to suppress their emotions to protect women and not intensify the woman's suffering after a pregnancy loss, because for most men her suffering was more legitimate. This was also reinforced by their social context, especially by women. ^14^, ^29^, ^32^, ^40^ Consequently, there can be a complex tension between maintaining the supportive façade and expression of suffering.^40^, ^41^

For men, attempts to alleviate suffering included trying to eliminate, belittle, and deny suffering or even resigning themselves to it.^28^ This was evident in other studies, in which emotional denial or suppression was used to repress their suffering.^12^, ^13^ This contributed to isolation and distancing behaviors with their partner.^12^, ^32^ Maintaining this façade indirectly resulted in greater suffering over time.

### Implications for Care

It is increasingly evident that integrated care is important and necessary for the immediate and long‐term well‐being of parents. However, current guidelines are inconsistent^17^ and mainly focus on medical management rather than emotional support.^17^, ^42^ Moreover, recommendations only focus on specialized care and on the precise moment of the loss, without considering follow‐up.^42^ Sensitive and supportive care requires professionals to go beyond technical aspects and to not only consider the loss as a clinical problem.^17^ As described in the literature, men barely felt included in care, which meant having to seek other services to clarify their doubts about the pregnancy loss process.^11^, ^12^, ^13^ There is a need for stronger couple‐centered collaboration between specialty and primary care. Ethical caregiving goes beyond physical care of women and should also incorporate emotional support of both women and men. However, the current training, structures, and organizational culture can make this holistic approach difficult. Our results show the impact on men and the support figure they represent for women. Therefore, incorporating men into care can have long‐term implications for their well‐being.

Health care professionals should have a heightened sensitivity to recognize suffering in whatever form it is expressed. Nonetheless, the professional's ability to acknowledge and alleviate suffering depends to some extent on one's own maturity in relation to one's own suffering.^28^ Midwives have a key role in this care because of their frequent interaction with couples during pregnancy.^43^ Previous research highlighted the difficulty of midwives in caring for parents who have suffered a pregnancy loss.^44^, ^45^ Specifically, primary care midwives did not know how to empathize with men or how to deal with their feelings.^45^ All of this can contribute to uncaring situations, in which emotional outreach to women and men is not prioritized.^44^, ^45^

### Limitations and Strengths

Although some believe that shared interviews could condition the participants’ responses, in our experience, individual discourse was found to be enhanced, and, in most cases, women acted as promoters of men's discourse. Socially, the suffering caused after an early loss is often less accepted, forcing those who experience it to negotiate its importance and to legitimize its existence. However, previous literature^46^ and our results suggest that gestational age was not a determining factor of distress after pregnancy loss. The inclusion of losses at different weeks’ gestation, and with different associated factors, such as the existence of previous children or difficulties in conception, provided a broad view of men's experiences. One participant had to decide with his partner to abort after the diagnosis of fetal anomalies incompatible with life. Despite the voluntary nature of the loss, it has been included because the decision was made from the certainty that the pregnancy was not going to be saved.

The homogeneity of the sample in terms of cultural context, sexual orientation, and relationship status were also limitations. Therefore, more research is required in other cultural contexts, including meta‐ethnographic studies, and samples should be expanded to include gay, bisexual, and transgender men, as well as single men who have separated after pregnancy loss.

## CONCLUSION

Pregnancy loss led to suffering in men, which they masked both in the meaning they gave to the loss and in social expression. To ensure holistic care, the suffering of men must be recognized and addressed. The theory of double‐layer masking of suffering could be used as a theoretical basis for future studies and for reflection and dialogue on caregiving that considers the needs of men after pregnancy loss.

## CONFLICT OF INTEREST

The authors have no conflicts of interest to disclose.
